# Supplementary material for: Tolerability of daily intermittent or continuous short-arm centrifugation during 60-day 6o head down bed rest (AGBRESA study)
Source: PLoS One. 2020 Sep 18;15(9):e0239228. doi: 10.1371/journal.pone.0239228 (PMC7500599; doi:10.1371/journal.pone.0239228)
Supplement: Attachment — Submitted filename: Response to Reviewers.docx [file pone.0239228.s005.docx]

**Response to Reviewer**

Dear Reviewer,

Dear Editor,

Thank you for your helpful comments on our manuscript. We have read them carefully and worked on a revised version that hopefully complies to your requirements. Please find a list of changes based on your input and comments below:

**Editor comments:**

1. Please provide an amended Funding Statement declaring this commercial affiliation, as well as a statement regarding the Role of Funders in your study. If the funding organization did not play a role in the study design, data collection and analysis, decision to publish, or preparation of the manuscript and only provided financial support in the form of authors' salaries and/or research materials, please review your statements relating to the author contributions, and ensure you have specifically and accurately indicated the role(s) that these authors had in your study. You can update author roles in the Author Contributions section of the online submission form.
2. Please also provide an updated Competing Interests Statement declaring this commercial affiliation along with any other relevant declarations relating to employment, consultancy, patents, products in development, or marketed products, etc.
3. Please include captions for your Supporting Information files at the end of your manuscript, and update any in-text citations to match accordingly.

**Answer**:

1.-2.: We have changed all declared parts including funding statement, author’s contribution and competing interests as requested.

3. We have added captions and a short description of all Supporting Information files.

**Reviewer#1 comments:**

1. The authors evaluate tolerability and acceptability of intermittent VS continuous short-arm centrifugation during long term head down bed rest. They conclude that both are tolerated, continuous a bit less. The manuscript is well written but I have a lot of concerns. First a control group is mentioned but not reported. I would say that before continuous Vs intermittent, the tolerability of daily centrifugation was the main question.

**Answer:** Thank you for pointing this out. Since daily centrifugation has not previously been tested in a longer-term bedrest study, we were concerned that adverse effects, such as syncope, could limit the utility of the intervention with increasing deconditioning and volume changes. If so, the countermeasure would be difficult to conduct during longer term space missions. Thus, from a practical point of view, the main question was how many centrifugation sessions would have to be aborted. Because the control group did not undergo centrifugation, there cannot be an aborted centrifugation. However, some of the more subjective tolerability assessments could be overestimated without adjustment of these measures in the control group. Unfortunately, these measurements were not obtained in the control group due to complexity of the study. We included the issue as potential limitation in the discussion (see lines 261-262).

2. By the way, tolerability /acceptability, can you explain the difference? Acceptability is not defined in the methods section.

**Answer:** We have changed the terminus to tolerability as this is much clearer.

3. 1G was set at the center of mass. Why? why not HIL, vestibular level, center of bones mass ? How was the center of mass determined ?

**Answer:** The centrifuge profile was a priori decided by NASA and ESA together with the DLR centrifuge science team. Previous centrifuge studies such as the BRAG1 study at MEDES (see Clement et al. 2015) showed good tolerability and partially effectiveness of both protocols during 5 days head down tilt bed rest. We have added a brief explanation in line 198-200. The setting in MEDES for BRAG1 was CoM at approximately 1.18 – 1.22 m from the CoR (Center of Rotation). The resulting rotational speed was 27.8 – 28.2, resulting in a +1Gz at CoM and approx. +2Gz at the feet. During AGBRESA we determined the center of mass of the subjects using anthropometry. Rotational speed of the centrifuge was therefore calculated for each subject individually. We also calculated and changed the position of each subject along the centrifuge radius to meet +2Gz at feet accordingly (see lines 112-120).

4. Pain is usually evaluated through 10 cm visual analog scale, this a especially important for repeated evaluation to prevent memorisation of the previous score. why did you choose a 0 to 20 scale which could potentially blur longitudinal scoring ?

**Answer:** We recorded motion sickness using a 0 to 20 scale (0 “I am feeling fine” to 20 “I am about to vomit”) that was fast and easy to use during centrifugation according Young et al. 2003. Pain was recorded only by the attending physician when reported by the subject and was not part of our experiment. As reported pain from other experiments (e.g. previous muscle biopsy) caused premature stops of intervention.

5. MSSQ: Your reference is that of Golding that is presumably concerning the MSSQ-short questionnaire, I think you should define MSA and MSB for non-specialists.

**Answer:** Thank you. We have added a short definition of MSA and MSB in the method section (see lines 138-140)

6. At some days it seems that the RPE scoring is higher (see for instance day 55) Can you comment on how other experiments conducted on these subjects (biopsy...) may have influenced tolerability ?

**Answer:** We have checked RPE scoring and the documentation of participant’s comments on pain during centrifugation after muscle biopsy experiments and have added this point to the discussion (lines 241-244).

**Reviewer#2 comments:**

7. This report appears to be the first of a number of reports that are based on the AGBRESA study, in which many research groups have studied various aspects of space deconditioning and the effects of artificial gravity as a countermeasure. In order to make these data available in a sound way, too many and too thin “salami” papers should be avoided. This reviewer therefore requests an over-all publication plan (confidential, not to be included in the paper).

**Answer:** ESA and NASA selected principal investigators for the AGBRESA study using a peer review approach. Each principal investigator has the right to publish experiments conducted as part of the AGBRESA study, however, there are some differences between NASA and ESA regulations. There is no overall-publication plan.

8. The term AGBRESA should be included such that it can be searched (abstract? title?), thereby allowing readers to get the full picture of the AGBRESA results.

**Answer:** In accordance with the comment above we have included the term AGBRESA also in the title.

9. SPECIFIC (line numbers introduced by the reviewer- should have been there in the first place)

40: during daily?

**Answer:** Corrected, thank you.

104-105 and Fig 1: Is it the area under the curves or the times at top spin rate that are identical between the continuous and the intermittent AG?

**Answer:** Due to the study design the times at top spin rate were identical, so both groups were exposed to daily 30 minutes AG with +1Gz at CoM. The intermittent group had a longer exposure to AG due to the frequent ramp up/down.

110: Radius at the foot plate? (could be computed by the reader from the spin rate, but that should not be necessary)

**Answer:** We calculated an individual centrifuge speed and position along centrifuge radius to ensure +1Gz at CoM and +2Gz at the foot plate. These radii were within 1729-2113 mm (see lines 112-120). E. Mulder et al. will publish a more general method paper elsewhere to give further details.

162-163: Foot pain has been reported in earlier AG studies.
177: p=0.67? tendency?

**Answer:** We changed the sentences to be more precise.

217: Is ref # 21 really relevant here? Do you mean #19? Missing original reference?

**Answer:** You are right, we changed the relevant reference.

256: Promissory notes regarding future studies are discouraged. Please rephrase.

**Answer:** As suggested, we rewrote the section.

**Reviewer #3 comments:**

1. A lot of text is provided about SANS. You neither measure it nor discuss your results in the context of SANS. I would suggest that you expand the introduction along the lines of the responses that you measured (hemodynamic, vestibular, etc.). Additional references should be placed in. For instance, Iwase et al., 2002; Goswami et al., 2015a; White et al., 2015)

**Answer:** Thank you. We have added missing sentences and references in the introduction.

2. Throughout the paper, no mention is made about gender. Then suddenly in the study results, we observe that the study involved males and females. The authors should discuss in the introduction what is the current evidence about sex-based differences in responses in space and/or during AG exposure. See, for example, Evans et al., 2018. Then in the discussion, the results obtained here (sex differences in the responses) should be thoroughly discussed.

**Answer:** We reported gender distribution of the study in the method section (see line 101) and have added recent findings from Evans et al. 2018 in the introduction. We discussed internally a sex based analysis of our results but the limited sample size (n = 8 per group) do not allow well powered statistics about gender differences. This topic was therefore also discussed with ESA. For the two upcoming bed rest studies involving AG as intervention will use two groups with n = 12 participants each. We also concluded in the discussion that a mixed sex cohort is required to further investigate AG as potential countermeasure.

3. If you were asked by a researcher, which of these protocols to use for future AG studies, what would you recommend? This could be a clear message of the paper and should be discussed at length in the paper. Currently, it is not clear which AG protocol should be used in the future.

**Answer:** You are right. We have rewritten and added additional text in the discussion section.

4. Could you also speculate which of these AG protocol would you recommend in the future if additional countermeasures such as nutrition or exercise are also added? See Iwase et al., 2005.

**Answer:** We have rewritten the discussion to make the planned combination of AG+exercises more clear.

5. Why did some of your participants faint/ develop presyncopal symptoms during AG exposure? Have you tested what their known limit of AG tolerance was? Recent evidence suggests that people take different times to develop presyncope on the centrifuge (Goswami et al.2015b). You should discuss your results in context to this paper as well as come up with clear recommendations about which AG protocol to use based on your evidence as well as published work.

**Answer:** During the Baseline data collection phase of AGBRESA only two short familiarization sessions with centrifugation were allowed for each subject. Therefore individual AG tolerance testing as shown by Goswami et al. was unfortunately not allowed by the study setting. To the authors knowledge this topic is currently under discussion by the ESA bed rest expert team for both upcoming bed rest studies in MEDES and Planica.
